# Does body mass index affect survival and technique failure in patients undergoing peritoneal dialysis?

**DOI:** 10.12669/pjms.301.3807

**Published:** 2014

**Authors:** Aydin Unal, Murat Hayri Sipahioglu, Ismail Kocyigit, Ferhan Elmali, Bulent Tokgoz, Oktay Oymak

**Affiliations:** 1 Aydin Unal, Department of Nephrology, Erciyes University Medical School, Kayseri, Turkey.; 2 Murat Hayri Sipahioglu, Department of Nephrology, Erciyes University Medical School, Kayseri, Turkey.; 3 Ismail Kocyigit, Department of Nephrology, Erciyes University Medical School, Kayseri, Turkey.; 4 Ferhan Elmali, Department of Biostatistics, Erciyes University Medical School, Kayseri, Turkey.; 5 Bulent Tokgoz, Department of Nephrology, Erciyes University Medical School, Kayseri, Turkey.; 6 Oktay Oymak, Department of Nephrology, Erciyes University Medical School, Kayseri, Turkey.

**Keywords:** Albumin, Body mass index, Peritoneal dialysis, Survival, Technique failure

## Abstract

***Objective:*** To investigate effect of body mass index (BMI) on survival and technique failure in patients undergoing peritoneal dialysis (PD).

***Methods:*** In this retrospective study three hundred ninety-two consecutive patients undergoing peritoneal dialysis from September 1995 to January 2013 were included. Median PD duration was 53 (range: 4-189) months. Clinical outcomes were mortality and technique failure. Technique failure was defined as transfer to hemodialysis (HD) due to peritonitis, ultrafiltration failure, inadequate dialysis, exit-site and/or tunnel infection, and mechanical problems. Deaths within 3 months after transferring to HD were accepted as PD-related mortalities. The patient and technique survival rates were estimated using the Kaplan-Meier method. Mortality risks were analyzed using the multivariate Cox regression model in which we included (in a backward-wald manner) all the significant variables from the univariate analysis.

***Results: ***There were 164 (41.8%) deaths. Forty-six (11.7%) patients underwent renal transplantation whereas 132 (33.7%) patients were transferred to HD. The multivariate Cox regression analysis found that the patient survival rates were significantly associated with age, BMI, baseline serum creatinine and albumin levels, and total Kt/Vurea. All variables as potential risk factors for the patient survival were also assessed for technique survival in univariate analysis and technique survival rates were significantly associated only with BMI (*p*: 0.015).

***Conclusion:*** BMI was associated with unfavorable patient survival in PD patients.

## INTRODUCTION

In the general population, higher body mass index (BMI) is observed to be associated with the increased prevalence of cardiovascular disease and overall mortality.^1^ On the other hand, a high BMI is associated with better survival in hemodialysis (HD) patients.^2^^,^^3^ This condition has been suggested as a “risk factor paradox” or “reverse epidemiology” for cardiovascular disease in dialysis patients.^4^^-^^6^ Compared to HD population, there are few studies in peritoneal dialysis (PD) population, in which the relationship between BMI and outcomes was evaluated, because of the greater frequency of HD as a treatment modality of end-stage renal disease (ESRD). The results of these studies are conflicting. Johnson and co-workers demonstrated that obesity is a favorable prognostic factor on survival.^7^ Some studies performed in PD patients showed that being normal weight had a survival advantage over overweight.^8^ The others demonstrated that obesity was associated with worse PD outcomes such as mortality and technique failure.^9^

In this study, we aimed to investigate effect of BMI on patient and technique survival in patients undergoing peritoneal dialysis.

## METHODS

 In this retrospective study three hundred ninety-two consecutive patients undergoing PD from September 1995 to January 2013 were included. All patients were followed at the PD unit of the Erciyes University Nephrology Department. Patients that survived more than 90 days following initiation of PD were included in this analysis. The following exclusion criteria were applied: (1) patients <18 years at start of PD , (2) patients who recovered from renal failure and no longer required dialysis, (3) patients that had data missing. The system was twin-bag. CAPD prescription was 4 × 2 L exchanges unless dialysis inadequacy was indicated by any sign. Median PD duration was 53 (range: 4-189) months.

Clinical outcomes were mortality and technique failure. Technique failure was defined as transfer to HD due to peritonitis, ultrafiltration failure, inadequate dialysis, exit-site and/or tunnel infection, and mechanical problems. Deaths within 3 months after transferring to HD were accepted as PD-related mortalities.

We recorded baseline biochemical measures, including blood urea nitrogen (BUN), serum creatinine, calcium, phosphorus, serum albumin, total cholesterol, low-density cholesterol, high-density cholesterol, triglyceride, and hemoglobin and clinical parameters such as systolic and diastolic blood pressures and BMI. We also noted baseline demographic data such as age and gender, the cause of ESRD, and other clinical data including dialysate/plasma (4-h D/P) creatinine ratio at the fourth hour of a dwell, which was determined by the standard peritoneal equilibration test^10^, and dialysis adequacy parameters such as weekly total and peritoneal Kt/Vurea.

BMI was defined as weight in kilograms divided by height in square meters (kg/m^2^).

Statistical analysis was performed using SPSS version 16.0 (SPSS Inc., Chicago, Illinois, USA). Transplantation and technique failure were censored observations for the patient survival analysis. Deaths unrelated to technique failure and transplantation were censored observations for the technique survival analysis. The patient and technique survival was estimated using the Kaplan-Meier method. Mortality risks were analyzed using the multivariate Cox regression model in which we included (in a backward-wald manner) all the significant variables from the univariate analysis. A *p *value of <0.05 was considered significant.

## RESULTS

Table-I shows baseline demographic, clinical, and biochemical parameters of the patients. Mean age at the start of PD was 51.0 ± 13.9 years; 227 of the 392 patients were male. The most known cause of ESRD was diabetes mellitus and hypertension. 

During the follow-up period of 22697 patient-months, there were 164 (41.8%) deaths, of which 75 (45.7%) were classified as cardiovascular, 28 (17.2%) peritonitis, 12 (7.3%) non-peritonitis infection, 12 (7.3%) cerebrovascular disease, 5 (3.0%) malignancy, and 32 (19.5%) other causes**.** Median follow-up duration of patients who died was 44.5 (4.0-189.0) months. The estimation of overall patient survival was 92.5 months. Estimation of patient survival was 94.7%, 89.9%, 81.9%, 65.8%, 45.6%, 35.8%, and 29.5% at 1, 2, 3, 5, 8, 10, 13 years, respectively.

Forty-six (11.7%) patients underwent renal transplantation whereas 132 (33.7%) patients were transferred to HD, which resulted from peritonitis/exit-site and/or tunnel infection in 85 (64.4%), ultrafiltration failure/inadequate dialysis in 21 (15.9%), mechanical malfunction in 14 (10.6%), and other causes in 12 (9.1%) patients. Median follow-up duration of patients transferred to HD was 49.0 (5.0-125.0) months. The estimation of overall technique survival was 107 months**.** Estimation of technique survival was 96.9%, 91.8%, 85.1%, 70.9%, 50.6%, 40.9%, and 37.5% at 1, 2, 3, 5, 8, 10, 13 years, respectively.

Univariate and multivariate analyses were performed to identify the risk factor(s) related to patient survival. Table-II shows univariate and multivariate analysis of risk factors for the patient survival. The variables, including age, gender, BMI, blood urea nitrogen, serum creatinine, corrected serum calcium x phosphorus, serum albumin, all blood lipid parameters, hemoglobin, weekly total Kt/Vurea, peritoneal Kt/Vurea, D/Pcreatinine ratio, and systolic and diastolic blood pressures were examined in univariate analysis as potential risk factors for the 392 patients. Six of these factors were statistically significant (*P *< 0.05). The multivariate Cox regression analysis found that the patient survival rates were significantly associated with age, BMI, serum creatinine and albumin levels, and total Kt/Vurea.

All variables as potential risk factors for the patient survival were also assessed for technique survival in univariate analysis. Technique survival rates were significantly associated only with BMI (*p*: 0.015; RR: 1.05; 95% confidence interval (CI): 1.01-1.09).

## Discussion

In this study, patients undergoing PD at our institution were evaluated in terms of clinical outcomes, technique failure and mortality. In multivariate cox regression analysis, it was found that the patients’ survival rates were significantly associated with age, BMI, baseline serum creatinine and albumin levels and total Kt/Vurea. We particularly focused effect of BMI on the PD patients’ survival rate and mortality in this study.

**Table-I:** Baseline demographic, clinical, and biochemical parameters of the patients

*Parameter*	
Age (year)	51.0 ± 13.9
Male/female (%)	227 (57.9%)/165 (42.1%)
Cause of end-stage renal disease (%)	
Diabetes mellitus	138 (35.2)
Hypertension	59 (15.1)
Glomerulonephritis	36 (9.2%)
Polycystic kidney disease	16 (4.1)
Other	39 (9.9)
Unknown	104 (26.5)
Body mass index (kg/m^2^)	23.6 ± 4.2
Systolic blood pressure (mmHg)	147.2 ± 29.8
Diastolic blood pressure (mmHg)	92.1 ± 18.2
Blood urea nitrogen (mg/dL)	53.8 ± 19.2
Serum creatinine (mg/dL)	6.8 ± 3.0
Corrected calcium x phosphorus (mg^2^/dL^2^)	42.2 ± 14.6
Serum albumin (g/dL)	3.6 ± 0.6
Total cholesterol (mg/dL)	193.4 ± 49.6
Low-density cholesterol (mg/dL)	113.3 ± 39.8
High-density cholesterol (mg/dL)	48.4 ± 15.3
Triglyceride (mg/dL)	158.6 ± 105.4
Hemoglobin (g/dL)	10.4 ± 1.8
Weekly total Kt/Vurea	2.39 ± 0.85
Weekly peritoneal total Kt/Vurea	1.70 ± 0.40
Dialysate/plasma creatinine ratio	0.67 ± 0.13

**Table-II:** Univariate and multivariate analysis of risk factors for the patient survival

	*Univariate analysis*		*Multivariate analysis*	
*Risk factor*	*RR (95% CI)*	*p value*	*RR (95% CI)*	*p value*
Age	1.02 (1.01-1.04)	<0.001	1.02 (1.00-1.03)	0.020
Body mass index	1.07 (1.03-1.11)	<0.001	1.05 (1.01-1.09)	0.010
Baseline serum creatinine	0.94 (0.89-0.99)	0.030	0.91 (0.85-0.97)	0.004
Baseline serum albumin	0.66 (0.51-0.83)	0.001	0.66 (0.52-0.84)	0.001
4-h D/Pcreatinine ratio	4.51 (1.33-15.39)	0.016	-	-
Total Kt/Vurea	0.80 (0.65-0.99)	0.045	0.71 (0.57-0.90)	0.004

RR: relative risk, CI: confidence interval, D: dialysate, P: plasma

A high BMI is associated with increased cardiovascular disease and all-cause mortality in the general population according to current literatures,^1^^,^^11^ In contrast to these observations high BMI is seemed like better outcomes in dialysis patients.^12^^-^^14^ However, most studies in PD patients have reported similar inverse weight-mortality relation. For instance, a 1% difference in percentage lean body mass (LBM) was associated with a 3% change deaths in the CANUSA study.^15^ Similarly, McCusker et al found a significantly lower patient survival rate in patients with lower LBM.^16^ On the other hand, in another study performed by Johnson et al., high BMI was associated with survival advantage in the 43 PD patients. Authors speculated that maintaining a higher-than-average BMI to preserve "nutritional reserve" may help to reduce the mortality and morbidity rates associated with PD.^7^

Conversely, many studies in PD patients found no survival advantages for obesity or indicated a higher risk of death in obese patients. Aslam et al^17^ studied 104 PD patients with a high BMI (> 27) matched to a control group of 104 patients with normal BMI (20-27) for age, gender, presence of diabetes, and Charlson Comorbidity index. They found that there was no relationship between high BMI and survival rate in this study. A large study was performed including Australia and New Zealand population for evaluating effect of obesity on survival rate.^9^ They found that patient mortality rates had a steady increase in death-censored technique failure rates with high BMI rates; whereas the mortality risk was lowest for BMI values of approximately 20 kg/m2. Authors concluded that obesity at the commencement of renal replacement therapy is a significant risk factor for death and technique failure. Another large study which included 1675 HD and 1662 PD patients was performed to investigate relationship between BMI and survival rates.^18^ It was found that any survival advantage associated with obesity among chronic dialysis patients is significantly less likely for PD patients, compared to HD patients. Correspondingly, BMI was associated with unfavorable patient survival in patients undergoing PD in our center. 

As mentioned above, there are several conflicting results on this subjects which could be confusing. However, BMI does not differentiate muscle mass and adipose tissue. Thus, these studies could not interpret properly since high BMI might result from either increased fat tissue or increased muscle mass. Since high BMI patients with inferred high body fat tissue had higher prevalence of atherosclerotic conditions such as cerebral, coronary and peripheral vascular diseases.

This hypothesis was confirmed by Beddhu et al^19^ using 24-hour urinary creatinine (UCr) excretion as a measure of muscle mass for evaluating relationship between high BMI and survival considering with and without fat tissue. Authors suggested that high BMI patients with inferred high body fat have increased and not decreased cardiovascular mortality. Antunes et al^20^ performed anthropometry, bioimpedance and nutritional follow-up in HD patients. They showed that higher muscle mass on account of higher protein intake offered survival advantage in dialysis patients. However, these methods are not applicable in all centers routinely.

## CONCLUSION

We have observed that high BMI is associated with mortality and technique failure. This study also demonstrates that age, baseline serum creatinine and albumin levels and total Kt/Vurea were associated with patient survival rates. 

## Author contribution:

Aydin Unal designed the study, collected and analyzed the data, and wrote the paper; Murat Hayri Sipahioglu was involved in designing of the study and collection of data; Ferhan Elmali performed statistical analysis; Bulent Tokgoz and Ismail Kocyigit collected the data; Oktay Oymak did final editing of the manuscript.

## Notes

***Limitation of the study: ***We did not perform procedures to assess muscle mass, which is important as these are generally considered more accurate than BMI.
